# *Crystallographic Tool Box* (*CrysTBox*): automated tools for transmission electron microscopists and crystallographers

**DOI:** 10.1107/S1600576715017252

**Published:** 2015-10-21

**Authors:** Miloslav Klinger, Aleš Jäger

**Affiliations:** a Laboratory of Nanostructures and Nanomaterials, Institute of Physics of the ASCR, Na Slovance 2, 182 21 Prague 8, Czech Republic

**Keywords:** electron diffraction, automated analysis, transmission electron microscopy, high-resolution transmission electron microscopy

## Abstract

Three tools for an automated analysis of electron diffraction pattern and crystallographic visualization are presented. Firstly, *diffractGUI* determines the zone axis from selected area diffraction, convergent beam diffraction or nanodiffraction patterns and allows for indexing of individual reflections. Secondly, *ringGUI* identifies crystallographic planes corresponding to the depicted rings in the ring diffraction pattern and can select the sample material from a list of candidates. Both *diffractGUI* and *ringGUI* employ methods of computer vision for a fast, robust and accurate analysis. Thirdly, *cellViewer* is an intuitive visualization tool which is also helpful for crystallographic calculations or educational purposes. *diffractGUI* and *cellViewer* can be used together during a transmission electron microscopy session to determine the sample holder tilts required to reach a desired zone axis. All the tools offer a graphical user interface. The toolbox is distributed as a standalone application, so it can be installed on the microscope computer and launched directly from *DigitalMicrograph* (Gatan Inc.).

## Abstract 2

The *CrysTBox* software for electron diffraction analysis and visualization is presented. Among others, the software offers an automated zone axis determination using selected area diffraction, convergent beam diffraction and nanodiffraction patterns, ring diffraction evaluation, and interactive crystal visualization in both direct and reciprocal space.

## 1. Introduction   

Most of those who have come across the field of crystallography know how exciting answering the questions raised in this domain can be. Unfortunately, most of them also know how tedious it is to answer everyday elementary questions which may often look quite simple: Which material am I looking at and what is the sample orientation? How is the (102) plane situated in this orientation? How should I tilt my TEM (transmission electron microscope) holder to get the sample into the [101] zone axis? Many other questions could follow. *CrysTBox* (*Crystallographic Tool Box*) should answer those questions or help to reach the answers more quickly.

The answers are often hidden in the images provided by a TEM. Those images can be easily acquired; however, their correct interpretation may be far from straightforward. *CrysTBox* employs artificial intelligence and computer vision, resulting in tools which allow the vast majority of the input images to be analysed fully automatically in a very short time. After setting the sample material, one click and about half a minute is enough to obtain the zone axis from the spot diffraction image or to identify the rings in the ring diffraction pattern. In the case of spot diffraction, the crystal lattice orientation in the direct space can be visualized and the holder tilt leading to an arbitrary sample orientation can be calculated. The toolbox also provides an intuitive interface allowing the user to see, name and interpret various crystallographic problems.

There are a number of programs and scripts for the analysis of the spot diffraction (Mitchell, 2008*b* ▸; Li, 2014 ▸; Wu *et al.*, 2012 ▸; Stadelmann, 2004 ▸; Reid *et al.*, 2011 ▸; Belletti *et al.*, 2000 ▸; Lábár, 2005 ▸) and ring diffraction patterns (Mitchell, 2008*a* ▸; Zhang *et al.*, 2011 ▸; Li, 2007 ▸; Reid *et al.*, 2011 ▸). Most of the software, however, requires a non-negligable amount of user intervention. These programs require the user to perform some part of the analysis, to process some partial results or to localize the image features (diffraction spots or ring centre). The manual localization of the image features is typically time consuming and often inaccurate. Some tools overcome these drawbacks by using a certain amount of automation. An interesting and robust tool for ring diffraction analysis was described by Mitchell (2008*a* ▸). Localization of the ring centres is done *via* a Hough transform, which may be memory and computationally intensive in fully automatic mode. The same author presents a tool for analysis of spot diffraction patterns (Mitchell, 2008*b* ▸) employing a circular average centred on one of the diffraction spots. This is a very fast and elegant approach, which reduces the number of spots localized by the user to one. On the other hand, it is very sensitive to the localization of the central spot. In contrast, the presented approach reconstructs the reciprocal lattice using many detected spots distributed across the pattern.

Another interesting tool, *Auto SADP*, is presented by Wu *et al* (2012 ▸). It can detect diffraction spots in a very fast and robust way. Several brightest spots are used to measure *d* spacings and interplanar angles. The *d* spacings, however, are measured between individual pairs of diffraction spots. In contrast, the *CrysTBox* tool *diffractGUI* fits a regular lattice to tens of detected reflections across whole diffraction pattern, which results in a higher accuracy. It can also successfully detect textured diffraction discs [*e.g.* convergent beam diffraction (CBED) discs] and offers further analyses and interpretation such as fully automatic zone axis determination and reflection indexing.

Compared to other available software, *CrysTBox* offers a significantly higher degree of automation. Although the tools are designed to be as automated and robust as possible, all major steps of the analysis can be controlled by the user *via* the graphical user interface (GUI). The user can see all relevant partial results, adjust parameters and thereby intervene in the analysis if necessary.

The toolbox also includes a visualization tool, *cellViewer*, which provides an intuitive and interactive instrument for crystal visualization in direct and reciprocal space. There are a number of similar computer programs (Boudias & Monceau, 1998 ▸; Stadelmann, 2004 ▸; Reid *et al.*, 2011 ▸; Momma & Izumi, 2008 ▸; Ozawa & Kang, 2004 ▸). Although they may be better suited for certain tasks, they are not always so interactive, so intuitive or free of charge.

## 2. *CrysTBox* tools   

At present, there are three tools available in *CrysTBox*: *diffractGUI* for spot diffraction analysis, *ringGUI* for ring diffraction analysis and *cellViewer* for visualization. Other tools are about to be released (*e.g.* a tool for TEM sample thickness measurement using CBED in the two-beam condition). The whole toolbox is implemented in MATLAB (The MathWorks, Natick, MA, USA) and C (Mex). Each tool is equipped with a GUI, which allows for user-friendly interaction and export of partial or final results. The analysis tools (*diffractGUI* and *ringGUI*) can also summarize the results into an HTML report.

The tools are launched *via* *CrysTBox Server*, an application allowing startup and control of individual tool instances by the user or computer programs. Apart from the buttons launching the tools, *CrysTBox Server* offers a file browser and image previewer and it allows for launching the tools by external applications. When started, *CrysTBox Server* creates a text file (so-called command file) and periodically checks it for changes. Commands written into this file, either by the user or by an external application, can be used to launch individual tools, analyse specified images *etc*.

The tools typically require the user to state the sample material and the input image. The sample material is defined using a text file describing the unit cell (lattice parameters, lattice angles, positions of the atoms in the unit cell and their atomic numbers). Detailed specification can be found at the *CrysTBox* web page (see §6). The image formats processible by *CrysTBox* include native *DigitalMicrograph* (Gatan Inc., Pleasanton, CA, USA) files (DM3 and DM4) as well as all common image formats (JPG, PNG, TIF *etc*.).

### 2.1. *diffractGUI*: spot diffraction analysis   

**Figure 1:**
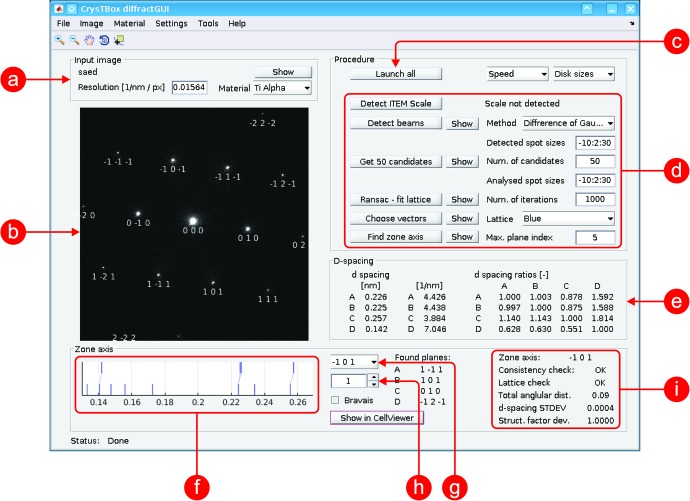
User interface of diffractGUI. (*a*) Input image details; (*b*) visualization of partial and final results in the input image; (*c*) button launching the whole analysis; (*d*) buttons launching individual steps of the analysis and basic settings; (*e*) parameters of the lattice found by RANSAC; (*f*) visualization of assignments between the theoretical *d* spacings (bottom) and experimental ones (top); (*g*) pop-up menu with possible solutions (zone axes); (*h*) calibration coefficient; (*i*) parameters of chosen solution.

This tool (see Fig.1 ▸) can be used especially for an automatic zone axis determination using diffraction or high-resolution TEM (HRTEM) images. The distances and angles between individual diffraction spots are quantified and the indices of corresponding planes are assigned to the spots. The whole procedure is immune to slight calibration inaccuracies. It can also be used to verify or fine-tune the camera scaling calibration.

#### 2.1.1. Input   

Apart from the information about the sample unit cell, an image is required. Input image types can include selected area diffraction (SAED), nanodiffraction or CBED patterns. An HRTEM image can also be used, as the tool automatically transforms it to the reciprocal space using a fast Fourier transform (FFT).

#### 2.1.2. Algorithm   

**Figure 2:**
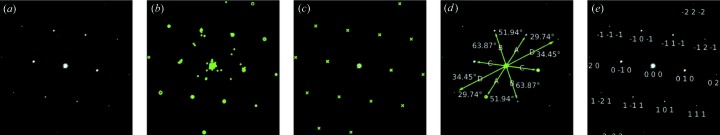
Individual steps of *diffractGUI* analysis. (*a*) Original image, (*b*) 100 strongest detections, (*c*) regular lattice found by RANSAC, (*d*) interplanar angles, (*e*) identified spots.

After the input image is loaded (Fig. 2 ▸ *a*), it may be beneficial to mask the beam stopper or burnt-in scale bars, if present, to prevent the diffraction spot detector from being confused. This can be done manually or automatically for certain beam stoppers.

The first crucial step of the analysis is the detection of diffraction spots or discs in the pattern. SAED or nanodiffraction spots are detected using blob detection in a scale space (Lowe, 2004 ▸) containing about 30 scales. The user can choose from different blob detection techniques: Gaussian detection, Hessian response or difference of Gaussians. In the case of a CBED pattern, the discs are detected using a Hough transform (Atherton & Kerbyson, 1999 ▸; Yuen *et al.*, 1990 ▸) as described by Klinger *et al.* (2015 ▸). The detected spots or discs are described using the centroid and score. The score is a measure reflecting the quality of a given detection. Since the blob detection method provides an enormous number of detections, many of which belong to the same diffraction spot, a non-maxima suppression is applied. This mechanism preserves only those detections whose score is locally maximal in the 26-connected neighbourhood. In other words, those detections whose neighbour (either within the image plane or in the scale space) has a higher score are ommited.

Since the detections can still suffer from a significant number of false positive or false negative detections, some intelligent mechanism must be used to extract a regular diffraction lattice from the detections. For this purpose, the RANSAC algorithm (Fischler & Bolles, 1981 ▸; Fischler & Bolles, 1987 ▸) is employed, as described by Klinger *et al.* (2015 ▸). It takes some number of the strongest detections (Fig. 2 ▸ *b*) as the input (50 highest scored candidates by default) and returns two vectors defining a regular lattice (Fig. 2 ▸ *c*). Parameters of this lattice (distances and angles between spots) are listed in the table (Figs. 1 ▸ *e* and 2 ▸ *d*) and may serve for a manual evaluation, for verification of the results or for other purposes. Knowledge of the sample unit cell provides sufficient information to compute theoretical *d*-spacing values and interplanar angles. The *d* spacings and interplanar angles measured in the lattice can be matched with the theoretical values lying in a certain range from the measured ones. Compared to a human, the computer can effectively examine a vast number of possible assertions of the theoretical values with respect to the experimentally measured ones and it can expeditiously verify whether a particular combination of planes corresponding to given *d* spacings and interplanar angles may be physically combined together to get a common zone axis.

The zone axes belonging to the solutions best fitting the experimental data are listed in a pop-up menu (Fig. 1 ▸ *g*) sorted according to an overall score reflecting differences between the experimental and theoretical *d* spacings and interplanar angles. When the user selects a particular zone axis, the indices of the planes corresponding to the diffraction spots are printed below the respective spot (Fig. 2 ▸ *e*), allowing for a straightforward interpretation of the experimental image.

#### 2.1.3. Features   

The most important benefit of this tool is the automation. The algorithm is designed to provide a fast and effortless analysis. Other advantages are precision and robustness. The RANSAC algorithm uses many spots distributed across the image to generate the regular lattice. Therefore, the result is more precise than multiple manual measurements between two selected diffraction spots. Moreover, the computer can see the spots that are not visible to the user owing to the very dynamic intensity range typical for the diffraction images.

The fact that the tool matches the measured values to the theoretical ones within some range around the measured values makes the whole procedure robust to some amount of camera calibration inaccuracy. Moreover, the tool can be used to find the correction coefficient for the camera length calibration.

In some cases, the tool can also be capable of processing two or three overlapping patterns, such as a diffraction pattern taken on interfaces (a twin matrix, adjacent grains separated by a grain boundary *etc*.). To find multiple lattices, the RANSAC algorithm is applied repeatedly while ignoring the spot detections close to the lattice points belonging to previously found lattices.

The results can also be passed to the interactive visualization tool *cellViewer* (§2.3).

### 2.2. *ringGUI*: ring diffraction analysis   

**Figure 3:**
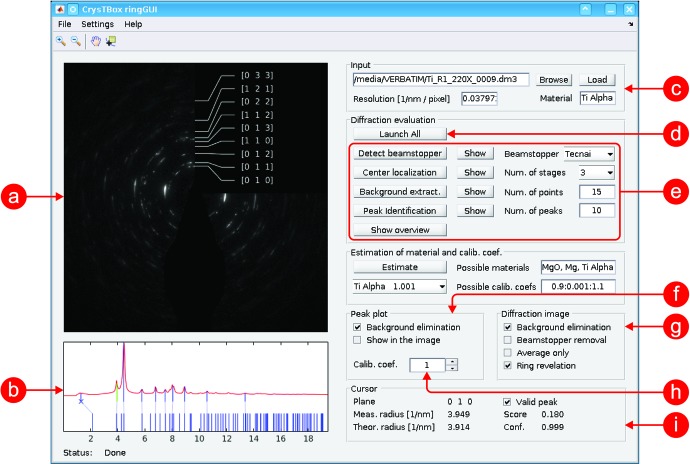
User interface of *ringGUI*. (*a*) Visualization of partial and final results in the input image; (*b*) visualization of assignment between measured peaks/rings (top) and theoretical *d*-spacing values (bottom); (*c*) input image details; (*d*) button launching the whole analysis; (*e*) buttons launching individual steps of the analysis procedure and basic settings; (*f*) settings of the peak plot; (*g*) instruments for enhancement of the input image; (*h*) calibration coefficient; (*i*) details about the peak/*d* spacing selected in the peak plot.

Ring diffraction patterns can be analysed using this tool (Fig. 3 ▸). It can automatically identify individual diffraction rings. If the sample material is not known precisely, the tool can select the best fitting one from the set of possible candidates given by the user. It also provides some image enhancement tools which help reveal the weakest reflections. Similarly to *diffractGUI*, it can also be used to find the correction of the camera length calibration.

#### 2.2.1. Input   

The input to the tool is the information about the sample unit cell and an experimental ring diffraction image. The ring intensity is not required to be uniform along the ring circumference. This tool can successfully process rings which are partially incomplete or which contain visible spots caused by a non-uniform grain sizing or preferred crystallite orientations. On the other hand, the rings should not be significantly distorted; for example, they should not be noticeably elliptical. In the case of a significant elliptical distortion, an appropriate correction should be performed prior to the analysis (Hou, 2008 ▸; Lábár, 2009 ▸).

#### 2.2.2. Algorithm   

Unlike the case for *diffractGUI*, the beam stopper must be eliminated here. Otherwise it would affect the mean intensity of the rings and the image background. The beam stopper can be masked manually or automatically in certain cases (based on an iterative thresholding). All the image pixels belonging to the beam stopper and its close neighbourhood are excluded from further processing.

In the next step, the centre of the rings needs to be localized. This is done by comparing the differences between the original input image and the images rotated around some candidate central point for 90, 180 and 270°. Each of the three rotated images is subtracted from the original one and the absolute value of this difference is averaged, yielding a measure of similarity between the rotated and original image. Finally, the three similarity measures corresponding to all three rotated images are averaged together, reflecting a fourfold rotational symmetry of the pattern with respect to the candidate central point. The candidate with the minimal mean difference (highest symmetry) is chosen as the ring centre.

When the ring centre is known, a circular mean can be calculated from the diffraction signal. The circular mean (or profile) is the mean image intensity as a function of the distance from the centre (see Fig. 3 ▸ *b*). Individual peaks on the profile correspond to the ring in the image.

Typically, there is some background in the image; the intensity tends to be higher in the central part of the pattern and fades outwards. This can be seen in the profile as well, so the profile can be used to quantify the background function. The background is approximated by a hyperbolic function. Its coefficients are iteratively optimized so that the hyperbola touches the profile from below.

After the background is subtracted from the profile, the peaks can be processed. Several highest peaks (ten by default) corresponding to the brightest rings are localized. Their distance from the centre is measured and compared with the theoretical ones specific to the sample material. Each experimental peak is crystallographically identified using the nearest theoretical one. In a similar manner to the *diffractGUI* tool, the *ringGUI* tool computes a camera length calibration value. For a material of known lattice parameter, this value can be used to calibrate the microscope or to routinely check the accuracy of existing calibrations.

If the sample material, camera length calibration or both are not exactly known, *ringGUI* can find the values best fitting the experimental data. The calibration coefficient can be specified as a range of feasible values, and the specimen material can be defined using a list of possible elements or compounds. The tool checks all possible combinations of calibration coefficients and materials and lists those which best fit the theoretical peaks.

#### 2.2.3. Features   

The main advantage of *ringGUI* is again the automation. Even very spotty images (Fig. 3 ▸) can be analysed quite quickly, easily and precisely.

**Figure 4:**
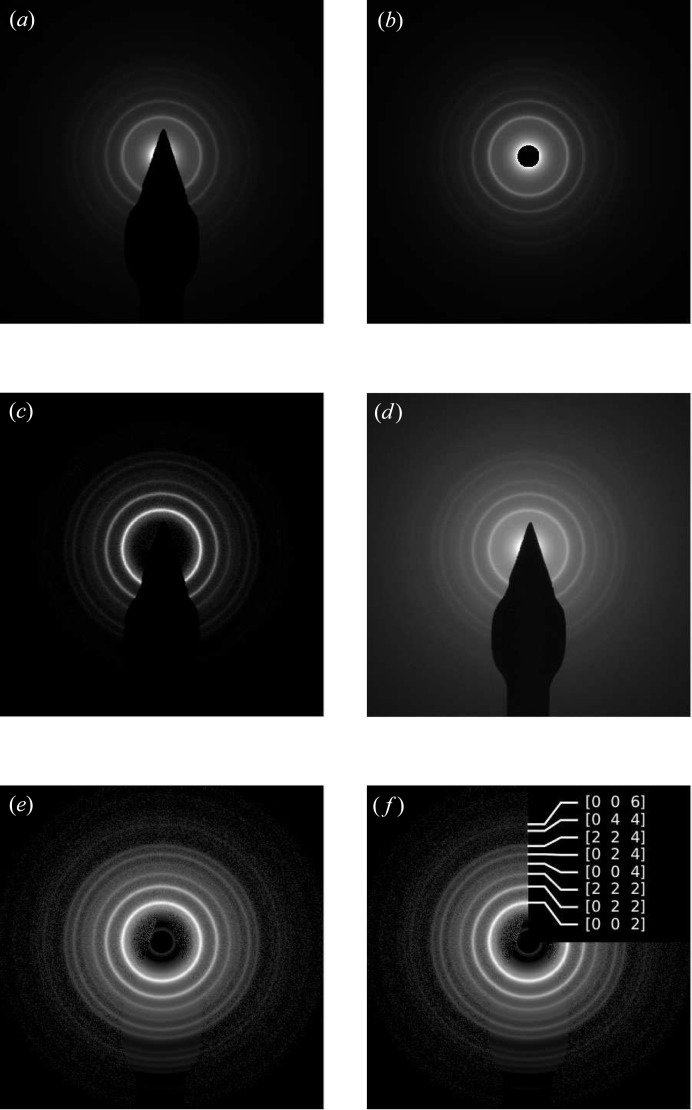
Image enhancement features of *ringGUI*. (*a*) Original image, (*b*) beam stopper removed, (*c*) background eliminated, (*d*) contrast adjustment, (*e*) combination of all, (*f*) rings identified.

Another great benefit is the image enhancement features. The shadow of the beam stopper can be removed from the original image (Figs. 4 ▸ *a* and 4 ▸ *b*). The area corresponding to the shadow is replaced with the circular average of intensities in the rest of the image. The background can be eliminated not only in the profile but also in the image (Fig. 4 ▸ *c*). The intensity contrast can be adjusted to make the weak rings more apparent (Fig. 4 ▸ *d*). Combined together, these instruments significantly improve the image readability (Figs. 4 ▸ *e* and 4 ▸ *f*).

The automatic determination of the calibration coefficient makes *ringGUI* a great asset in camera length calibration tasks.

### 2.3. *cellViewer*: visualization   

**Figure 5:**
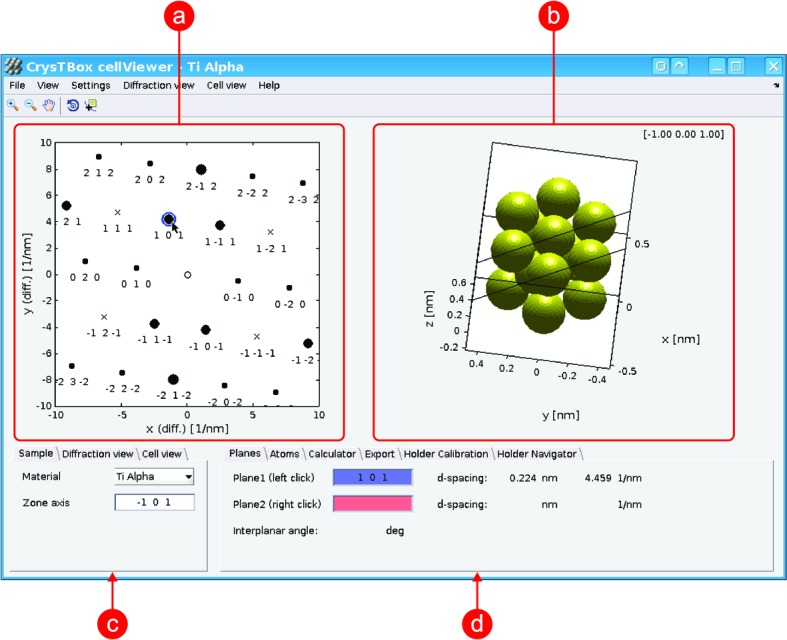
User interface of *cellViewer* – visualization of *diffractGUI* results as shown in Fig 1 ▸. (*a*) Diffraction view; (*b*) cell view; (*c*) basic settings of material and diffraction and cell view; (*d*) details about selected diffraction spots or atoms, calculator, and other utilities.

*cellViewer* (Fig. 5 ▸) provides an interactive view of the reciprocal and direct space. It can be used as a visualization tool, a crystallographic calculator or just an educational aid, allowing the user to play with certain crystallographic phenomena and to reveal how they are related to each other.

#### 2.3.1. Input   

The only required input is the unit-cell information. In a cooperation with *diffractGUI*, the tool can receive information about the experimental diffraction lattice in order to produce its exact theoretical counterpart and correspondingly oriented atomic lattice.

#### 2.3.2. Features   

There are two main views in *cellViewer*: the diffraction view containing a theoretical diffraction pattern (Fig. 5 ▸ *a*) and the cell view showing several atoms of a simulated sample (Fig. 5 ▸ *b*). Both views are interactive. As they often show the same thing from a different point of view, they are linked together, making the relations between the direct and reciprocal space more apparent. In the diffraction view, one can select up to two spots using a mouse click. Planes corresponding to those spots are instantly shown in the cell view in the context of the atomic structure (this can be seen in Figs. 5 ▸ *a* and 5 ▸ *b*). The simulated sample in the cell view can be zoomed or rotated. When rotating the sample, the diffraction view can stay intact, allowing the user to inspect the sample from different points of view. However, the diffraction pattern can also be instantly updated according to the actual orientation of the sample, immediately illustrating the effect of the sample rotation. The cell view is also interactive. The user can select one atom to get its coordinates and element, two atoms to get information about the corresponding direction vector, three atoms giving information about a given plane and its normal, and four atoms adding the *d*-spacing values.

*cellViewer* also provides a crystallographic calculator. Among others things, it offers conversion between the crystallographic coordinate system (in Miller indices) and the Cartesian coordinate system. It can calculate *d* spacing, angles between planes or vectors, atomic scattering amplitude, structure factor, and extinction distance. A text file containing a table of the selected quantities can be exported.

*cellViewer* can gain from the results of *diffractGUI* analysis. After *diffractGUI* has finished its analysis of an experimental diffraction image, it can pass the results to *cellViewer*. A theoretical diffraction pattern in the diffraction view and the orientation of the simulated sample in the cell view then fully correspond to the experimental image. This offers a very fast and intuitive way to see how the atoms are arranged in the crystal observed.

If the user provides basic information about the sample holder (such as the α and β tilt used to acquire a given experimental image or the direction of the α axis in the image), the holder tilt can be simulated [another such tool is described by Duden *et al.* (2011 ▸)]. As the user tilts the simulated sample, all relevant information is updated instantly; the simulated sample is tilted, the theoretical diffraction pattern is changed accordingly and the zone axis read-out is actualized. Moreover, the tool can calculate the α and β tilt needed to reach a desired zone axis.

## 3. Integration   

The tools can be used independently from one another; however, they are much more powerful if they cooperate with each other or with external applications. As mentioned above, *diffractGUI* can pass the results to *cellViewer* for visualization purposes or for navigation of the sample holder. All the tools can also be integrated with any application that is able to write into the command file. This is a standard text file periodically checked by *CrysTBox Server*, where the commands controlling the tools can be entered.

Of particular benefit is the integration with Gatan *DigitalMicrograph* (*DM*), which is a commonly used software for image acquisition and processing in transmission electron microscopy. It provides a very powerful scripting which makes the application quite flexible and customizable (Mitchell & Schaffer, 2005 ▸). Among other things, it allows for an easy installation of extensions *via DM* plugins. Thanks to the *DM* plugin distributed together with *CrysTBox*, the image analysis can be launched directly from *DM* in a single click. The delay between seeing the image on the microscope fluorescent screen and seeing the analysis results can therefore be reduced to about five clicks and one minute.

## 4. Computer *versus* human   

**Figure 6:**
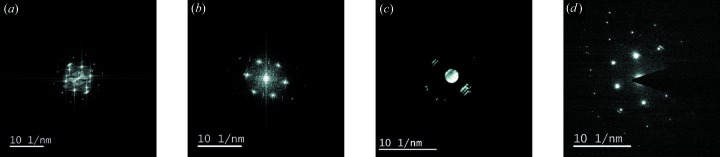
Diffraction patterns involved in the case study. (*a*) Image 1, Mg in [001] zone axis, (*b*) Image 2, γ-Ti in [1
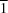
0] zone axis, (*c*) Image 3, Mg in [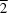
01] zone axis, (*d*) Image 4, Mg in [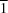
11] zone axis.

To demonstrate the efficiency and abilities of the automated tool, a small case study focused on evaluation of spot/disc diffraction patterns was performed. It involved four images: two HRTEM images transformed using FFT (Fig. 6 ▸ *a* and 6 ▸ *b*), one CBED pattern (Fig. 6 ▸ *c*) and one spot diffraction pattern (Fig. 6 ▸ *d*). The analysis objective was to index the spots/discs, measure corresponding *d* spacings and determine the zone axis. The images were evaluated by two experienced human analysts and by *diffractGUI* [tested on a Lenovo ThinkPad E540, Intel Core i7 (2.3/3.3 GHz) running on a single core]. The information provided to the analysts was the image in DM3 format, sample material and corresponding table of *d*-spacing values. Apart from that, they were allowed to use arbitrary literature or software with the exception of the software features performing the analysis or its parts automatically.

The zone axes were successfully determined except for Image 2, where Analyst 2 was not able to find the zone axis. Table 1 ▸ shows the theoretical and measured *d*-spacing values and corresponding deviations. Compared to the human analysts, the estimates of *diffractGUI* are slightly closer to the theoretical values on average. It can also be seen that the deviations within one image tend to be more consistent for *diffractGUI*. This consistency could indicate that the automated algorithm achieves a lower method-induced error. A significant difference can be seen in the time required for the analysis (Table 2 ▸). The results vary from image to image and analyst to analyst. Generally, it can be concluded that *diffractGUI* is 30–60 times faster than a human.

## 5. Availability   

Further information about the software can be found at the web page http://www.fzu.cz/CrysTBox or in the reference manual (Klinger, 2015 ▸). The site contains installation guides, system requirements, sample videos, video tutorials, and examples of input data and corresponding results. The specification of the file describing the sample unit cell is also available. The graphical user interface of each tool is described in detail and the parameters and settings are explained.

*CrysTBox* is available on-demand as a standalone application for the Windows platform. Restrictions imposed by MATLAB Compiler do not allow the software to be used for commercial purposes or by commercial subjects.

## 6. Conclusions   

*CrysTBox* makes electron diffraction analysis significantly faster, less tedious and more accurate. The analysis tools, *diffractGUI* and *ringGUI*, are especially notable for TEM users who deal with electron diffraction every day. The amount of automation opens a wide variety of possible applications, for instance in advanced instrumentation or materials characterization. The fact that the tools can be installed on the microscope computer, and, moreover, that they can cooperate with other computer programs, makes them an ideal teammate for an electron microscopist. The visualization and educational abilities of *cellViewer* further widen the range of the toolbox users.

Although tricky data will always exist, the vast majority of images can be analysed fully automatically. This especially holds for spot/disc diffraction analysis using *diffractGUI*. Therefore, the use of these tools can save a significant amount of time and effort. Even if some amount of human interaction is needed, the tools are still helpful as they allow for a comfortable and user-friendly control of the whole analysis procedure.

## Figures and Tables

**Table 1:** The *d*-spacing values (*d*-sp., in nm) measured in the four images in Fig. 6 ▸ by *diffractGUI* and two human analysts together with deviations (dev., in pm, differences between the theoretical and measured values) The average deviation was calculated from the absolute values of the deviations.

	Theoretical	*diffractGUI*		Analyst 1		Analyst 2	
	*d*-sp.	*d*-sp.	dev.	*d*-sp.	dev.	*d*-sp.	dev.
Image 1	0.2780	0.2762	−1.8	0.2732	−4.8	0.2747	−3.3
	0.2780	0.2750	−3.0	0.2747	−3.3	0.2738	−4.2
	0.2780	0.2783	0.3	0.2778	−0.2	0.2795	1.5

Image 2	0.2499	0.2481	−1.8	0.2469	−3.0	0.2490	−0.9
	0.2499	0.2482	−1.7	0.2487	−1.2	0.2480	−1.9
	0.2300	0.2279	−2.1	0.2294	−0.6	0.2293	−0.7

Image 3	0.1901	0.1924	2.3	0.1941	4.0	0.1900	−0.1
	0.2780	0.2802	2.2	0.2762	−1.8	0.2745	−3.5
	0.1901	0.1909	0.8	0.1866	−3.5	0.1901	0.0

Image 4	0.2453	0.2442	−1.1	0.2439	−1.4	0.2402	−5.1
	0.2453	0.2444	−0.9	0.2445	−0.8	0.2443	−1.0
	0.1901	0.1862	−3.9	0.1866	−3.5	0.1873	−2.8

Average			1.8		2.3		2.1

**Table 2:** Time required to analyse the four images in Fig. 6 ▸, together with speed-ups offered by *diffractGUI* in comparison with humans

	*diffractGUI*	Analyst 1		Analyst 2	
	Time (s)	Time (s)	Speed-up (×)	Time (s)	Speed-up (×)
Image 1	22	480	22	1200	55
Image 2	12	390	33	2700	225
Image 3	20	980	49	1320	66
Image 4	17	650	38	960	56
Average			36		100
